# Stapedius reflex testing shows altered small muscle function in untreated Pompe patients and improvement after enzyme replacement therapy

**DOI:** 10.1186/1471-2474-14-S2-P5

**Published:** 2013-05-29

**Authors:** M Hilz, U Hoppe, S Moeller, J Köhn

**Affiliations:** 1 Department of Neurology, University of Erlangen-Nuremberg, Erlangen, Germany; 2 Departments of Neurology, Medicine, Psychiatry, New York University, New York, NY, USA; 3 Deparment of Audiology, University of Erlangen-Nuremberg, Erlangen, Germany

## Introduction

Pompe disease primarily affects skeletal and cardiac muscles. It is difficult to assess therapeutic efficacy of enzyme replacement therapy (ERT) using primary end points based on changes in weakness of large limb-girdle muscles, gait-endurance, and respiratory function, which all depend on variable day-to-day patient performance. In contrast, muscle improvement might be shown by assessing function of very small muscles, such as the stapedius and tensor tympani muscles. We therefore tested whether ERT benefits in Pompe patients can be shown by stapedius reflex testing before and after ERT.

## Results

In four Pompe patients, we assessed ipsilateral stapedius reflex thresholds in the right and left ear one and two years after patients received first dosage of biweekly ERT with alglucosidase alfa (Myozyme™, 20 mg/kg KG i.v.). In two patients, stapedius reflex thresholds were also assessed before ERT-onset. Muscle tension of the stapedius and tensor tympani muscles were determined by measuring acoustic impedance at the tympanic membrane in response to single bursts of 0.5, 1, 2 and 4 kHz tones. Reflex thresholds between 70 and 90dB HL are within the normal range; higher responses indicate impaired thresholds. Patient 1 (female, 46 years) had no stapedius reflex responses before ERT-onset, reflex thresholds of 98.8/87.5dB (right/left; R/L) after one year of ERT, and reflex thresholds of 91.3/87.5dB (R/L) after two years of ERT. Patient 2 (male, 65 years) had reflex thresholds of 98.8/93.8dB (R/L) before ERT-onset, reflex thresholds of 86.3/91.3dB (R/L) after one year of ERT, and reflex thresholds of 90.0/91.3dB (R/L) after two years of ERT. Patient 3 (female, 53 years) had reflex thresholds of 93.8dB (R) and no stapedius reflex responses (L) after one year of ERT, and reflex thresholds of 96.3/98.8dB (R/L) after two years of ERT. Patient 4 (female, 69 years) had reflex thresholds of 85.0/85.0dB (R/L) after one year of ERT, and reflex thresholds of 80.0/81.3dB (R/L) after two years of ERT.

## Conclusion

In untreated Pompe patients, stapedius reflex testing demonstrates impaired small muscle function. After one and two years of ERT, stapedius reflex thresholds improved. Consequently, stapedius reflex testing can objectively demonstrate significant improvement of muscle function with ERT.

